# Necrotising pneumonia caused by *Curvularia hawaiiensis* (syn. *Bipolaris hawaiiensis*) and *Mycobacterium tuberculosis* coinfection in a patient with ascariasis: a case report and review

**DOI:** 10.1186/s12941-023-00593-z

**Published:** 2023-05-13

**Authors:** Cristina Aguirre, Jaime David Acosta-España, Sheila Jissela Patajalo-Villata, Alfonso J. Rodriguez-Morales

**Affiliations:** 1 School of Medicine, Universidad Internacional del Ecuador, Quito, Ecuador; 2 Department of Internal Medicine, Hospital Vozandes Quito, Quito, Ecuador; 3 Institute of Microbiology, Friedrich Schiller University Jena, Beutenbergstraße 13, 07745 Jena, Germany; 4 Postgraduate Program in Infectious Diseases, School of Medicine, Pontificia Universidad Católica del Ecuador, Quito, Ecuador; 5 Graduate Program in Internal Medicine, Pontificia Universidad Católica del Ecuador, Quito, Ecuador; 6 Master of Clinical Epidemiology and Biostatistics, Universidad Científica del Sur, Lima, Peru; 7 Gilbert and Rose-Marie Chagoury School of Medicine, Lebanese American University, P.O. Box 36, Beirut, Lebanon

**Keywords:** Necrotising pneumonia, *Curvularia hawaiiensis* (syn. *Bipolaris hawaiiensis*), Tuberculosis, Ascariasis, Coinfection

## Abstract

**Introduction:**

*Curvularia hawaiiensis* (formerly *Bipolaris hawaiiensis*) is a plant pathogen often isolated from soil and vegetative material. However, only a few cases of opportunistic invasive infections in humans have been described.

**Case:**

A 16-year-old female patient without comorbidities was admitted to the emergency department because of fever and chest pain. We described the first coinfection of *Curvularia hawaiiensis* and *Mycobacterium tuberculosis* necrotising pneumonia.

**Discussion:**

Multiple infections can alter immune responses. However, immunosuppression is the most critical risk factor for infection with species of the genus *Curvularia*. Therefore, it is crucial to carefully examine patients with tuberculosis, as they may rarely be coinfected with unusual fungi.

**Supplementary Information:**

The online version contains supplementary material available at 10.1186/s12941-023-00593-z.

## Background

Patients with respiratory symptoms may be infected with bacteria, viruses, fungi or parasites. In 2021, tuberculosis (TB) incidence was estimated at 10,600,000 cases, with 309,000 cases in the Americas [1]. In Ecuador, at least 8500 cases were estimated for 2021 [2]. Fungi infect the lungs of patients with risk factors such as immunodeficiency, chronic diseases and malignancies, to name a few [3]. A study in Iran described that 12.3% of the studied population had coinfection with fungi and TB [4]. *Curvularia hawaiiensis* is mainly associated with allergic bronchopulmonary diseases. No cases of necrotising pneumonia or coinfection with TB were found in the literature reviewed (PubMed, Embase, ClinicalKey, ScienceDirect, Scopus and WoS). Therefore, we report a case of coinfection with TB and *C. hawaiiensis* in a patient with ascariasis and discuss its implication.

## Case

A 16-year-old female patient, born and living in Colón, Esmeraldas, Ecuador. Family history: grandfather with high blood pressure (HBP), maternal grandmother with HBP, diabetes, breast cancer and sister with pleural effusion of unknown aetiology. Surgical history of umbilical herniorrhaphy 15 years ago. The patient took an unspecified medication to treat a diagnosed urinary tract infection two weeks before this hospitalisation (first week of November 2021).

On 23 November 2021, the patient was admitted to the emergency department complaining of a moderately pressing headache, mild oppressive retrosternal pain and dyspnoea at rest for 24 h. There was also an unquantified temperature increase, hyporexia and asthenia for two weeks.

On physical examination, blood pressure was 109/63 mmHg, heart rate 126 bpm, respiratory rate 28 min, temperature 35.9 °C, oxygen saturation 90% with 0.5 L O_2_ via nasal cannula. Weight 49 kg and height 1.62 m with a body mass index of 18.7 kg/m^2^. Conscious, oriented, dehydrated, diaphoretic patient. Dry oral mucosa, dry lower lip with thrush. Persistent chest expansion, tachypnoeic. Lung auscultation showed absent breath sounds in the left lung base and decreased air entry in the right lung base with fine crackles. Abdomen increased air-fluid sounds.

**Fig. 1:**
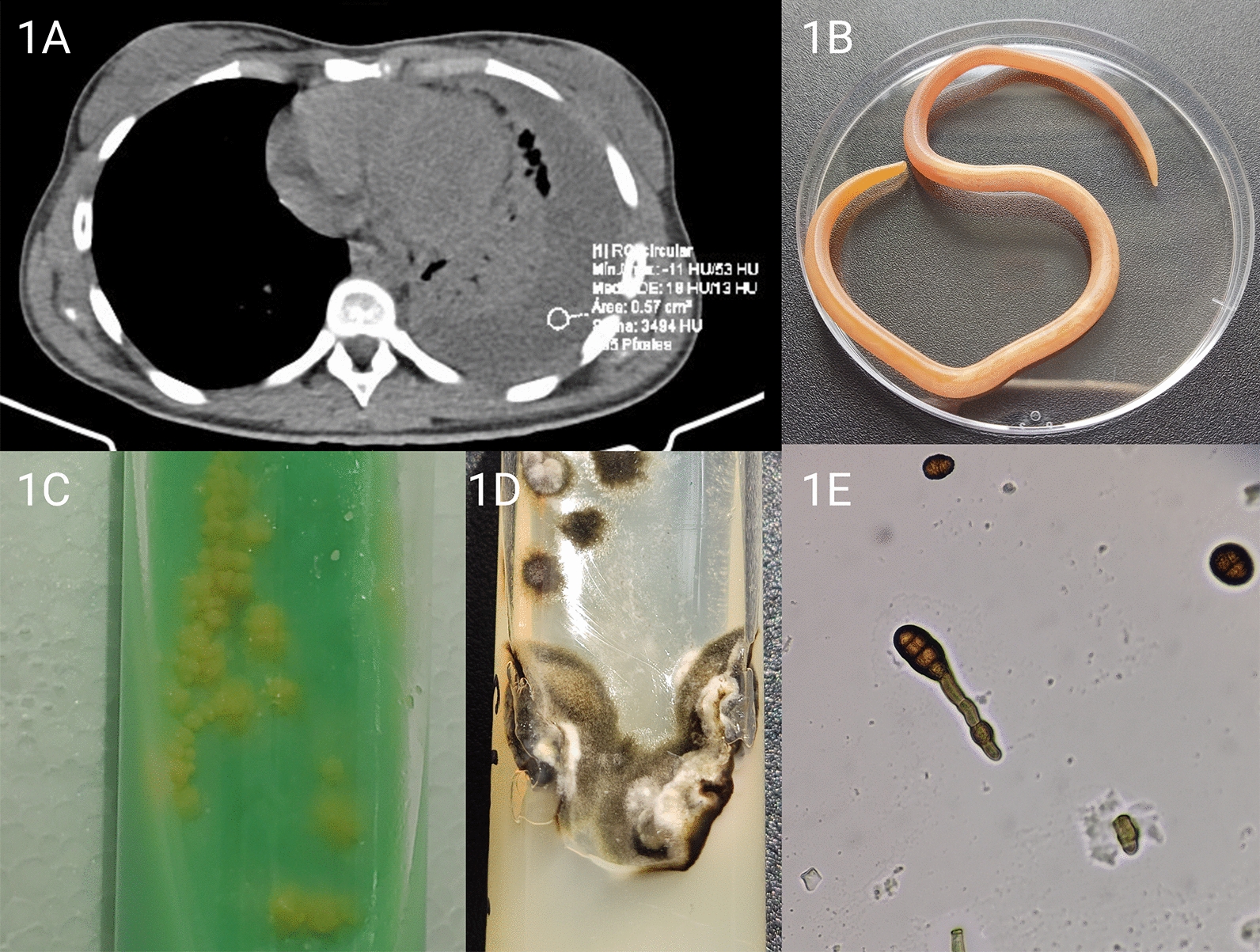
Relevant radiological and microbiological findings of the case. **A** Thoracic tomography shows pleural effusion in the left hemithorax, approximate volume 485 mL, maximum density 38 hu, parietal and visceral pleura thickening, and mediastinal calcifications. Left anterior apical calcified granuloma, subpleural laminar and segmental atelectasis left basal consolidation with aerial bronchogram. Pre-aortic and subcarinal reactive nodules. Small right pericardial effusion. **B** Female helminth of *A. lumbricoides* obtained from the patient's vomit. **C** *Mycobacterium tuberculosis* isolated from lung biopsy on Lowenstein Jensen agar. **D** Culture of a lung biopsy on Sabouraud agar with chloramphenicol showing filamentous growth of *Curvularia hawaiiensis*. **E** Microscopic view of *C. hawaiiensis* ha colony with ellipsoid conidia, rounded at the ends, pale brown, medium reddish brown to dark brown, three septa

Based on the clinical findings, chest tomography, complete blood count, C-reactive protein, procalcitonin, erythrocyte sedimentation rate, lactate dehydrogenase (LDH), d-dimer, ferritin, serum iron, liver profile, blood biochemistry, electrolytes, coagulation, hormones, and lipid profile were requested (Table 1). Tests for human immunodeficiency virus infection were negative on two separate tests. Thoracic tomography (Fig. 1A) showed a pleural effusion in the left hemithorax, an approximate volume of 485 mL, and a maximum density of 38 hu.

**Table 1:** Lab results of the case

White blood cell count	Normal range	Pre-treatment						Post-treatment	
		23/11	24/11	26/11	27/11	28/11	30/11	12/21	04/22
Leucocytes u/L	4.8–10.8	6.4	4.39	4.9	14.12	13.32	10.02	5.76	4.32
Neutrophils u/L	2.06–7.02	4.65	3.0	2.41	12.77	10.23	6.44	3.35	1.58
Lymphocytes u/L	0.98–4.91	1.07	0.89	1.77	0.97	2.25	2.91	1.58	2.18
Monocytes u/L	0.09–0.97	0.58	0.36	0.5	0.34	0.79	0.87	0.59	0.38
Eosinophils u/L	0.05–0.54	0.03	0.09	0.17	0	0.02	0.21	0.17	0.12
Neutrophils %	43.0–65.0	72.6	68	49.2	90.4	76.8	64.2	58.2	36.5
Lymphocytes %	20.5–45.5	16.7	20.3	36,1	6.9	16.9	20.1	27.4	50.5
Monocytes %	1.9–9.0	9.1	8.2	10.2	2.4	5.9	8.7	10.2	8.8
Eosinophils %	1.0–5.0	0.5	2.1	3.5	0	0.2	2.1	3.0	2.8
Haemoglobin g/dL	12.1–16.2	8.7	8.4	8.3	10.6	9.5	10.9	12.6	13.9
Hematocrit %	40.0–51.0	29.4	28,1	28	32.5	28.4	35.7	41.1	44.4
Mean corpuscular volumen fL	80.0–100.0	79.7	79.6	79.5	78.7	77.4	79.7	80.0	79.9
Mean corpuscular haemoglobin pg	27.0–31.0	23.6	23.8	23.6	25.7	25.9	24.3	24.5	25
Platelet count	100.000–500.00	441	433	509	466	494	646	438	297
Reticulocytes %		0.95	0	0				0	0
Erythrocyte sedimentation rate	0.0–10.0	107							
Serology									
C-Reactive Protein (CRP) mg/L	0.0–5.0	168.28		174.79		139.28	177.18		
Procalcitonin (PCT) ng/mL	2.0	0.28							
Lactic Dehydrogenase (LDH) U/L	135.0–250.0	282							
d Dimer ug/mL	0.0–0.5	> 5.0							
Ferritin ng/mL	13.0–150.0	814.9							
Iron ug/dL	37.0–147.0	18							
Blood chemistry tests									
Creatinine mg/dL	0.5–0.9	0.63			0.51				
Total protein g/dL	6.4–8.3	7.23							
Albumin g/dL	3.4–4.8	3.18							
Globulin g/dL	2.1–3.2	4.05							
Total Bilirubin mg/dL	0.0–1.1	0.31							
Direct Bilirubin mg/dL	0.0–0.3	0.17							
Indirect Bilirubin mg/dL	0.0–7.0	0.15							
Liver enzymes									
AST U/L	0.0–32.0	16.2						18.5	25.3
ALT U/L	0.0–33.0	13.2						11	13.7
Coagulation profile									
TP seg		13.8							
TTP seg		33.3							
INR		1.31							
Lipid profile									
Total cholesterol mg/dL		102							
Electrolytes									
Sodium mmol/L	136.0–145.0				135	140			
Potassium mmol/L	3.5–5.5				4.3	4.4			
Hormones									
Thyroid stimulating hormone UI/mL	0.51–4.3		1.95						
Pleural fluid									
Leucocytes u/mm^3^		581							
Red blood cells u/mm^3^		2490							
Mononuclear %		97							
Polymorphonuclear %		3							
Glucose mg/dL		50.1							
Protein g/dL		5.6							
Cholesterol mg/dL		60.4							
DHL U/L		735

On 24 November 2021, it was decided to perform a pleural puncture, during which 120 mL of yellow-green fluid was obtained. Examination of the pleural fluid (Table 1) revealed the presence of leukocytes with 97% monocytes, erythrocytes and LDH 735. In addition, Ziehl–Neelsen staining was negative for mycobacteria, adenosine deaminase was negative, and amplification of nucleic acids by GeneXpert MTB/RIF detected *Mycobacterium tuberculosis*. Based on these results, an administrative procedure was initiated with the Ecuadorian Ministry of Health to obtain antituberculous therapy. On the same day, the patient experienced spontaneous vomiting at night, and according to the laboratory report, a female *Ascaris lumbricoides* was detected (Fig. 1B).

On 25 November 2021, antibiotic therapy was started with ampicillin plus sulbactam 3 grammes intravenously every 6 h daily for seven days. In addition, albendazole 400 mg orally was introduced for one dose.

On 25 November 2021, fever and chest pain persisted despite intravenous antipyretic medication at therapeutic doses. Therefore, cardiothoracic surgery performed thoracoscopy, complete lung decortication, parietal pleurectomy and wedge-shaped lung resection. Lung and pleural biopsy showed growth of *M. tuberculosis* in a culture that responded to first-line antituberculosis drugs (Fig. 1C). In addition, *Curvularia hawaiiensis* (Fig. 1D, E), was isolated from a fungal culture of lung tissue and identified by sequencing the internal transcribed spacer and D1/D2 regions of the rDNA (7 December 2021).

On 26 November 2021, oral antituberculosis therapy was started with isoniazid (245 mg), pyrazinamide (1225 mg), ethambutol (735 mg) and rifampicin (490 mg) for two months. And 6 months were completed with rifampicin and isoniazid.

The patient was treated with itraconazole 200 mg twice daily for 16 weeks. Monthly follow-ups were done for the first three months, and the patient showed no progression of her infections. One year ago, there were no relapses or new hospitalisations at the follow-up.

## Discussion

Despite the joint efforts of the World Health Organisation (WHO) and the governments of various countries, the 2025 milestones of the WHO End TB strategy have not been achieved. In 2015, for example, reducing TB incidence by 50% was one of the critical global targets, but only 10% was achieved from 2015 to 2021 [5]. In Ecuador, directly supervised short-term therapy (DOTS) was introduced in 2000 to identify and promptly treat TB rapidly. However, this strategy has suffered several setbacks, such as constant staff turnover [6].

Although the incidence of TB in Ecuador showed a clear downward trend between 2000 and 2005. From 2006 to 2007, it remained stable, with an incidence of about 40 cases per 100,000 population (pop). Finally, from 2017 to 2021, a slight increase, with 48 cases per 100,000 pop [7]. In Ecuador, TB remains endemic. And among the provinces with high prevalence is Esmeraldas, where the patient reported in this case lives.

Active pneumonia TB may be associated with other pathogens. Among fungal infections in patients with TB, the most commonly reported microorganisms are *Candida* sp., *Aspergillus* sp., *Histoplasma capsulatum* and *Cryptococcus neoformans* [4, 8]. In addition, it is essential to mention the sum of risk factors in fungal infections. For example, patients with *Aspergillus fumigatus* and cystic fibrosis colonisation are associated with high mortality and an unfavourable prognosis [9]. The patient studied, in this case, had no underlying disease. However, based on the calcifications seen on tomography (Fig. 1b), contact with TB was likely prolonged. And according to some reports, active TB becomes a risk factor for fungal infections in certain previously healthy patients [8, 10].

Our patient's only known risk factor was active pulmonary and extrapulmonary tuberculosis, with a recent initial diagnosis. With the isolation of *Curvularia hawaiiensis* by selective fungal culture from a thoracoscopically obtained lung biopsy. As far as we know, and after reviewing the primary scientific databases, no association of *Curvularia* sp. or *C. hawaiiensis* has been reported in patients with active TB worldwide. Only 18 cases of *C. hawaiiensis* infection are in the literature (Additional file 1). Of these, 4 cases of allergic bronchopulmonary disease (ABD) showed productive cough, dyspnoea and elevated blood eosinophil levels. Clinically, our patient presented on admission with dyspnoea, pressing retrosternal chest pain and moderate headache. That is different from cases of *C. hawaiiensis* in patients with ABP. All cases of ABP due to *C. hawaiiensis* were treated with corticosteroids plus antifungals (amphotericin B lipid complex and itraconazole) [11, 12] in two cases and potassium iodide in two cases [13]). Therapy is not well established, so this case was treated with 200 mg itraconazole twice daily for 16 weeks, and no relapses or hospitalisations were observed.

Tuberculosis remains a significant disease in many endemic areas. Despite concerted international efforts, the "WHO End TB Strategy" goals could not be achieved. For this reason, screening for TB is always recommended in areas with a high incidence of TB cases. In addition, patients with active TB may be at increased risk for fungal infections in certain patients. We report the first case of confirmed infection with *Curvularia hawaiiensis* and active pulmonary and extrapulmonary TB plus gastrointestinal ascariasis and moderate anaemia in a young patient without other previous comorbidities.

Regarding fungal colonisation, in patients with chronic respiratory diseases, *Aspergillus* and *Candida* species have been found [14]. However, this is not the case. A clinical presentation does not characterise colonisations; these are usually incidental findings in patients with good general health [14]. Acute tuberculosis is uncommon, and calcified lung lesions on CT show infection, inconsistent with acute disease. Generally, patients with tuberculosis form tissue lesions that develop into granulomas. In chronic patients, these develop into cavities [15].

This patient does not have chronic inflammatory disease, immunosuppression or other causes that increase the risk of colonisation. And tuberculosis does not explain the necrosis and lung damage observed in the study CT. This patient required surgical exploration by thoracoscopy. And a lung biopsy culture under sterile conditions remains the diagnostic reference tool [16]. The clinical condition, the radiological lesions, the surgical solution with necrotic lung tissue and the positive, pure culture obtained by the microbiological service under sterile conditions suggest pulmonary coinfection by *Curvularia hawaiiensis* (syn. *Bipolaris hawaiiensis*) and *Mycobacterium tuberculosis*. Accurate clinical and laboratory diagnosis is critical for managing these patients, in whom various coinfections may be overlooked.

## Supplementary Information

**Additional file 1.** Systematic review *Curvularia hawaiiensis* case reports.

## Data Availability

Not applicable.

## Abbreviations

ABD: Allergic bronchopulmonary disease
DOTS: Directly Observed Therapy Short Course
HBP: High blood pressure
hu: Hounsfield unit
LDH: Lactate dehydrogenase
Tb: Tuberculosis
WHO: World Health Organization

## References

[1] World Health Organization. Tuberculosis. https://www.who.int/news-room/fact-sheets/detail/tuberculosis (2022).

[2] World Health Organization. Tuberculosis profile. https://worldhealthorg.shinyapps.io/tb_profiles/?_inputs_&lan=%22EN%22&entity_type=%22country%22&iso2=%22EC%22 (2021).

[3] Phoompoung P (2022). Risk factors of invasive fungal infections in lung transplant recipients: a systematic review and meta-analysis. J Heart Lung Transplant.

[4] Amiri MRJ, Siami R, Khaledi A (2018). Tuberculosis status and coinfection of pulmonary fungal infections in patients referred to Reference Laboratory of Health Centers Ghaemshahr City during 2007–2017. Ethiop J Health Sci.

[5] World Health Organization. *Global Tuberculosis Programme*. https://www.who.int/teams/global-tuberculosis-programme/tb-reports (2022).

[6] Houston S (2004). Tuberculosis control in Ecuador: unforeseen problems, unanticipated strengths. Can Respir J.

[7] Incidence of tuberculosis (per 100,000 people) - Ecuador | Data. World Health Organization, Global Tuberculosis Report. https://data.worldbank.org/indicator/SH.TBS.INCD?locations=EC (2021).

[8] Hosseini M, Shakerimoghaddam A, Ghazalibina M, Khaledi A (2020). Aspergillus coinfection among patients with pulmonary tuberculosis in Asia and Africa countries; a systematic review and meta-analysis of cross-sectional studies. Microb Pathog.

[9] Speirs JJ, van der Ent CK, Beekman JM (2012). Effects of *Aspergillus fumigatus* colonisation on lung function in cystic fibrosis. Curr Opin Pulm Med.

[10] Njovu IK (2021). Status of pulmonary fungal pathogens among individuals with clinical features of pulmonary tuberculosis at Mbarara University Teaching Hospital in Southwestern Uganda. Ther Adv Infect Dis.

[11] Chowdhary A (2011). *Bipolaris hawaiiensis* as etiologic agent of allergic bronchopulmonary mycosis: first case in a paediatric patient. Med Mycol.

[12] Saenz RE, Brown WD, Sanders CV (2001). Allergic bronchopulmonary disease caused by *Bipolaris hawaiiensis* presenting as a necrotising pneumonia: case report and review of literature. Am J Med Sci.

[13] Mcaleer R, Kroenert DB, Elder JL, Froudist JH (1981). Allergic bronchopulmonary disease caused by *Curvularia lunata* and *Drechslera hawaiiensis*. Thorax.

[14] Biswas D, Agarwal S, Sindhwani G, Rawat J (2010). Fungal colonisation in patients with chronic respiratory diseases from Himalayan region of India. Ann Clin Microbiol Antimicrob.

[15] Bhalla AS, Goyal A, Guleria R, Gupta AK (2015). Chest tuberculosis: radiological review and imaging recommendations. Indian J Radiol Imaging.

[16] Miller JM (2018). A guide to utilisation of the Microbiology Laboratory for Diagnosis of Infectious Diseases: 2018 Update by the Infectious Diseases Society of America and the American Society for Microbiology. Clin Infect Dis.

