# Supplementary material for: Necrotising pneumonia caused by Curvularia hawaiiensis (syn. Bipolaris hawaiiensis) and Mycobacterium tuberculosis coinfection in a patient with ascariasis: a case report and review
Source: Ann Clin Microbiol Antimicrob. 2023 May 13;22:36. doi: 10.1186/s12941-023-00593-z (PMC10183113; doi:10.1186/s12941-023-00593-z)
Supplement: Supplementary file 1 — Additional file 1. Systematic review Curvularia hawaiiensis case reports. [file 12941_2023_593_MOESM1_ESM.docx]

**Systematic review *Curvularia hawaiiensis* case reports**

**Method: PRISMA 2020**^1^

**Author:** Jaime David Acosta-España, M.D., M.Sc.

**Taxonomy Browser**

Taxonomy ID: 1230527

homotypic synonym: *Bipolaris hawaiiensis*, *Cochliobolus hawaiiensis*, *Pseudocochliobolus hawaiiensis*.

**Index Fungorum**

Identifier 800543

**PubMed: 2 results**

**Terms:** *Curvularia hawaiiensis*

**Filter:** case report

**Inclusion criteria:** case report with isolate of *Curvularia hawaiiensis*

**Exclusion criteria:** Manuscripts other than case reports of human infections. Not having microbiological confirmation of Curvularia hawaiiensis.

Time period: 1981 – 16/11/2022

- Corneal ulcer due to a rare pleosporalean member of the genus *Bipolaris* (B. *australiensis)* following cow tail injury to the eye: A case report and review of literature

Link: <https://pubmed.ncbi.nlm.nih.gov/28573998/>

Citation: Pai HV, Jamal E, Yegneswaran PP. Corneal ulcer due to a rare pleosporalean member of the genus Bipolaris following cow tail injury to the eye: A case report and review of literature. Indian J Ophthalmol. 2017 May;65(5):403-405. doi: 10.4103/ijo.IJO_836_16. PMID: 28573998; PMCID: PMC5565883.

- Allergic bronchopulmonary disease caused by Curvularia lunata and ***Drechslera hawaiiensis***

Link: <https://pubmed.ncbi.nlm.nih.gov/7314001/>

McAleer R, Kroenert DB, Elder JL, Froudist JH. Allergic bronchopulmonary disease caused by Curvularia lunata and Drechslera hawaiiensis. Thorax. 1981 May;36(5):338-44. doi: 10.1136/thx.36.5.338. PMID: 7314001; PMCID: PMC471506.

**Pubmed: 17 results**

**Terms:** *Bipolaris hawaiiensis*

**Filter:** case report

**Inclusion criteria:** case report with isolate of Bipolaris hawaiiensis

**Exclusion criteria:** Manuscripts other than case reports of human infections. Not having microbiological confirmation of *Bipolaris hawaiiensis*. Previously included cases are excluded to avoid duplication.

Time period: 1986 – 16/11/2022

**Results**

- ***Cochliobolus hawaiiensis*** Sinusitis, a Tropical Disease? A Case Report and Review of the Literature

Link: <https://pubmed.ncbi.nlm.nih.gov/25805318/>

Gautier M, Michel J, Normand AC, Cassagne C, Piarroux R, Ranque S. Cochliobolus hawaiiensis Sinusitis, a Tropical Disease? A Case Report and Review of the Literature. Mycopathologia. 2015 Aug;180(1-2):117-21. doi: 10.1007/s11046-015-9886-1. Epub 2015 Mar 25. PMID: 25805318.

- Subcutaneous phaeohyphomycosis caused by *Bipolaris hawaiiensis* in an immunocompetent patient

Link: <https://pubmed.ncbi.nlm.nih.gov/25382522/>

Verma R, Roy P, Vasudevan B, Bhatt P, Kharayat V, Kaur G. Subcutaneous phaeohyphomycosis caused by *Bipolaris hawaiiensis* in an immunocompetent patient. Indian J Dermatol Venereol Leprol. 2014 Nov-Dec;80(6):554-6. doi: 10.4103/0378-6323.144198. PMID: 25382522.

- *Bipolaris hawaiiensis* as etiologic agent of allergic bronchopulmonary mycosis: first case in a paediatric patient

Link: <https://pubmed.ncbi.nlm.nih.gov/21395476/>

Chowdhary A, Randhawa HS, Singh V, Khan ZU, Ahmad S, Kathuria S, Roy P, Khanna G, Chandra J. *Bipolaris hawaiiensis* as etiologic agent of allergic bronchopulmonary mycosis: first case in a paediatric patient. Med Mycol. 2011 Oct;49(7):760-5. doi: 10.3109/13693786.2011.566895. Epub 2011 Mar 14. PMID: 21395476.

- *Bipolaris hawaiiensis* keratomycosis and endophthalmitis

Link: <https://pubmed.ncbi.nlm.nih.gov/18622717/>

Bashir G, Hussain W, Rizvi A. *Bipolaris hawaiiensis* keratomycosis and endophthalmitis. Mycopathologia. 2009 Jan;167(1):51-3. doi: 10.1007/s11046-008-9144-x. Epub 2008 Jul 12. PMID: 18622717.

- Subungual hyperkeratosis of the big toe due to *Bipolaris hawaiiensis*

Link: <https://pubmed.ncbi.nlm.nih.gov/15844642/>

Romano C, Ghilardi A, Massai L. Subungual hyperkeratosis of the big toe due to Bipolaris hawaiiensis. Acta Derm Venereol. 2004;84(6):476-7. PMID: 15844642.

- Invasive fungal sinusitis due to *Bipolaris hawaiiensis*

Link: <https://pubmed.ncbi.nlm.nih.gov/14998405/>

Castelnuovo P, De Bernardi F, Cavanna C, Pagella F, Bossolesi P, Marone P, Farina C. Invasive fungal sinusitis due to Bipolaris hawaiiensis. Mycoses. 2004 Feb;47(1-2):76-81. doi: 10.1046/j.0933-7407.2003.00941.x. PMID: 14998405.

- Allergic bronchopulmonary disease caused by *Bipolaris hawaiiensis* presenting as a necrotizing pneumonia: case report and review of literature

Link: <https://pubmed.ncbi.nlm.nih.gov/11269801/>

Saenz RE, Brown WD, Sanders CV. Allergic bronchopulmonary disease caused by *Bipolaris hawaiiensis* presenting as a necrotizing pneumonia: case report and review of literature. Am J Med Sci. 2001 Mar;321(3):209-12. doi: 10.1097/00000441-200103000-00012. PMID: 11269801.

- Allergic fungal sinusitis caused by *Bipolaris* (**Drechslera)** *hawaiiensis*

Link: <https://pubmed.ncbi.nlm.nih.gov/10473824/>

Fryen A, Mayser P, Glanz H, Füssle R, Breithaupt H, de Hoog GS. Allergic fungal sinusitis caused by *Bipolaris (Drechslera) hawaiiensis*. Eur Arch Otorhinolaryngol. 1999;256(7):330-4. doi: 10.1007/s004050050157. PMID: 10473824.

- Peritoneal dialysis complicated by *Bipolaris hawaiiensis* peritonitis: successful therapy with catheter removal and oral itraconazol without the use of amphotericin-B

Link: <https://pubmed.ncbi.nlm.nih.gov/7573196/>

Gadallah MF, White R, el-Shahawy MA, Abreo F, Oberle A, Work J. Peritoneal dialysis complicated by *Bipolaris hawaiiensis* peritonitis: successful therapy with catheter removal and oral itraconazol without the use of amphotericin-B. Am J Nephrol. 1995;15(4):348-52. doi: 10.1159/000168862. PMID: 7573196.

- Endogenous endophthalmitis caused by *Bipolaris hawaiiensis* in a patient with acquired immunodeficiency syndrome.

Link: <https://pubmed.ncbi.nlm.nih.gov/8238229/>

Pavan PR, Margo CE. Endogenous endophthalmitis caused by *Bipolaris hawaiiensis* in a patient with acquired immunodeficiency syndrome. Am J Ophthalmol. 1993 Nov 15;116(5):644-5. doi: 10.1016/s0002-9394(14)73211-8. PMID: 8238229.

- Subcutaneous phaeohyphomycosis caused by *Bipolaris hawaiiensis*. A case report

Link: <https://pubmed.ncbi.nlm.nih.gov/1843402/>

Costa AR, Porto E, Tabuti AH, Lacaz Cda S, Sakai-Valente NY, Maranhão WM, Rodrigues MC. Subcutaneous phaeohyphomycosis caused by *Bipolaris hawaiiensis*. A case report. Rev Inst Med Trop Sao Paulo. 1991 Jan-Feb;33(1):74-9. doi: 10.1590/s0036-46651991000100014. PMID: 1843402.

*Bipolaris hawaiiensis*-caused phaeohyphomycotic orbitopathy. A devastating fungal sinusitis in an apparently immunocompetent host.

Link: <https://pubmed.ncbi.nlm.nih.gov/2704535/>

Maskin SL, Fetchick RJ, Leone CR Jr, Sharkey PK, Rinaldi MG. *Bipolaris hawaiiensis*-caused phaeohyphomycotic orbitopathy. A devastating fungal sinusitis in an apparently immunocompetent host. Ophthalmology. 1989 Feb;96(2):175-9. doi: 10.1016/s0161-6420(89)32917-4. PMID: 2704535.

Corneal ulcer caused by Bipolaris hawaiiensis

Link: <https://pubmed.ncbi.nlm.nih.gov/3236148/>

Anandi V, Suryawanshi NB, Koshi G, Padhye AA, Ajello L. Corneal ulcer caused by Bipolaris hawaiiensis. J Med Vet Mycol. 1988;26(5):301-6. PMID: 3236148.

- Nasal phaeohyphomycosis caused by *Bipolaris hawaiiensis*.

Link: <https://pubmed.ncbi.nlm.nih.gov/3325631/>

Koshi G, Anandi V, Kurien M, Kirubakaran MG, Padhye AA, Ajello L. Nasal phaeohyphomycosis caused by *Bipolaris hawaiiensis*. J Med Vet Mycol. 1987 Dec;25(6):397-402. PMID: 3325631.

- Granulomatous encephalitis caused by *Bipolaris hawaiiensis*

Link: <https://pubmed.ncbi.nlm.nih.gov/3778150/>

Morton SJ, Midthun K, Merz WG. Granulomatous encephalitis caused by *Bipolaris hawaiiensis*. Arch Pathol Lab Med. 1986 Dec;110(12):1183-5. PMID: 3778150.

- Emerging agents of phaeohyphomycosis: pathogenic species of *Bipolaris* and *Exserohilum*

Link: <https://pubmed.ncbi.nlm.nih.gov/3745423/>

McGinnis MR, Rinaldi MG, Winn RE. Emerging agents of phaeohyphomycosis: pathogenic species of *Bipolaris* and *Exserohilum*. J Clin Microbiol. 1986 Aug;24(2):250-9. doi: 10.1128/jcm.24.2.250-259.1986. PMID: 3745423; PMCID: PMC268884.

**Pubmed: 6 results**

**Terms:** *Cochliobolus hawaiiensis*

**Filter:** case report

**Inclusion criteria:** case report with isolate of *Cochliobolus hawaiiensis*

**Exclusion criteria:** Manuscripts other than case reports of human infections. Not having microbiological confirmation of *Cochliobolus hawaiiensis*. Previously included cases are excluded to avoid duplication.

Time period: 1986 – 16/11/2022

**Results**

The articles were already included authors: Gautier M, Verma R, Chowdhary A, Bashir G, Castelnuovo P, Saenz RE. Duplication was avoided with triple verification

**Pubmed: 0 results**

**Terms:** Pseudocochliobolus hawaiiensis

**Filter:** case report

**Inclusion criteria:** case report with isolate of *Pseudocochliobolus hawaiiensis*

**Exclusion criteria:** Manuscripts other than case reports of human infections. Not having microbiological confirmation of *Pseudocochliobolus hawaiiensis*. Previously included cases are excluded to avoid duplication.

Time period: 1986 – 16/11/2022

**Results**

No results.

**Reference**

1. Page MJ, McKenzie JE, Bossuyt PM, et al. The PRISMA 2020 statement: an updated guideline for reporting systematic reviews. *Syst Rev*. 2021;10(1):1-11. doi:10.1186/S13643-021-01626-4/FIGURES/1
